# Multi-Epitope Peptide-Based and Vaccinia-Based Universal Influenza Vaccine Candidates Subjected to Clinical Trials

**DOI:** 10.21315/mjms2020.27.2.2

**Published:** 2020-04-30

**Authors:** Syazwani Romeli, Sharifah Syed Hassan, Wei Boon Yap

**Affiliations:** 1 Biomedical Science Programme, Faculty of Health Sciences, Universiti Kebangsaan Malaysia, Kuala Lumpur, Malaysia; 2 Jeffrey Cheah School of Medicine & Health Sciences, Monash University Malaysia, Selangor, Malaysia; 3 Center of Toxicology and Health Risk Studies, Faculty of Health Sciences, Universiti Kebangsaan Malaysia, Kuala Lumpur, Malaysia

**Keywords:** conserved region, influenza virus, multi-epitope peptide, universal vaccine, vaccination

## Abstract

In light of the limited protection conferred by current influenza vaccines, immunisation using universal influenza vaccines has been proposed for protection against all or most influenza sub-types. The fundamental principle of universal influenza vaccines is based on conserved antigens found in most influenza strains, such as matrix 2, nucleocapsid, matrix 1 and stem of hemagglutinin proteins. These antigens trigger cross-protective immunity against different influenza strains. Many researchers have attempted to produce the conserved epitopes of these antigens in the form of peptides in the hope of generating universal influenza vaccine candidates that can broadly induce cross-reactive protection against influenza viral infections. However, peptide vaccines are poorly immunogenic when applied individually owing to their small molecular sizes. Hence, strategies, such as combining peptides as multi-epitope vaccines or presenting peptides on vaccinia virus particles, are employed. This review discusses the clinical and laboratory findings of several multi-epitope peptide vaccine candidates and vaccinia-based peptide vaccines. The majority of these vaccine candidates have reached the clinical trial phase. The findings in this study will indeed shed light on the applicability of universal influenza vaccines to prevent seasonal and pandemic influenza outbreaks in the near future.

## Introduction

Influenza is an acute respiratory infection caused by influenza viruses. The World Health Organization (WHO) estimates that 3–5 million cases of influenza occur each year and the infection accounts for 250,000–500,000 deaths worldwide (1). Vaccination is the best and the most cost-effective measure for reducing the impact of influenza. However, current seasonal influenza vaccines confer limited protection at only between 10% and 60%, which is lower than the protection that most licensed non-influenza vaccines can ever provide (2). The mismatch of vaccine strains with circulating influenza strains is the main reason for the suboptimal vaccine effectiveness (3). On top of that, predicting the strains that must be included in the formulation of vaccines for combating upcoming influenza outbreaks is difficult owing to gene mutations through antigenic drift and shift patterns in influenza viruses (4). Moreover, the use of hemagglutinin (HA) (5) as the antigen of choice in the present vaccine formulation and the classic manner of producing influenza vaccine in embryonated chicken eggs reduce the efficacy of influenza vaccines (6). HA protein functions as receptor-binding antigen and has high plasticity. It undergoes amino acid changes to facilitate virus replication during egg adaptation (7). As a result, its antigenicity is altered and the stimulation of humoral responses that inhibit the binding of virus to host cells is limited.

These obstacles have led to the idea of developing universal influenza vaccines. Such vaccines can provide durable protection for all age groups against infections by all influenza sub-types (seasonal and pandemic) or at least broadly protect against a number of such sub-types over several seasons (2). Basically, universal influenza vaccines are designed and developed based on conserved viral antigens amongst influenza strains: matrix 2 (M2), nucleocapsid (NP), matrix 1 (M1), polymerase basic 1 (PB1) and stem of hemagglutinin (HA2) proteins (8–9). These conserved antigens can elicit the production of antibodies that recognise homosubtypic and heterosubtypic strains and subsequently provide protection against them. In response to upcoming human influenza outbreaks and pandemics, the development of universal influenza vaccines has gained increasing interest and has been greatly advocated. For instance, Tan et al. (10) expressed nonstructural 1 (NS1) protein on the cell wall of *Lactobacillus casei (L. casei)*. NS1 protein is conserved in the majority of influenza virus, and its presence on the cell wall of *L. casei* did not alter its antigenicity. Thus, this protein is believed to hold great potential as an oral universal influenza vaccine. In spite of various endeavours in the development of universal influenza vaccines, no definitive front-runner of universal influenza vaccine is available. However, a number of candidates that have been subjected to clinical trials, such as multi-epitope peptide and vaccinia-based vaccines, are available.

## Universal Influenza Vaccine Candidates

### Multimeric-001

Multimeric-001 (M-001) is an example of synthetic peptide vaccine that is produced based on nine conserved immunogenic epitopes from HA, NP and M1 proteins of influenza type A and B strains. These epitopes are known to induce humoral and cellular immune responses (11). The development of peptide vaccines through a chemical approach allows the synthesis of specific epitopes that can induce targeted immune responses. Given the mode of synthesis, chemically synthesised peptides are relatively stable and free from any hazardous effect (12). However, given their small molecular sizes, they are poorly immunogenic and hence require carriers to improve their efficacy (13). The efficacy of M-001 as an anti-influenza vaccine can be enhanced by initially expressing the epitopes individually on the flagellin protein of *Salmonella* which provides both carrier and adjuvant functions (11). Flagellin has been widely used as a carrier and is known to be safe and able to increase the immunogenicity of vaccines in several animal models (14). Interferon gamma (IFN-γ) secretion is higher when chemically synthesised peptides are presented on flagellin than when flagellin carriers are absent. In addition, flagellin prolongs the exposure of peptides to the mouse immune system. Without flagellin, peptides degrade rapidly within a few minutes after being administered intramuscularly (15).

The immunogenicity of the peptides displayed individually on flagellin was validated in mice challenged with influenza A virus (IAV) H2 and H3 subtypes. One of them was a B cell epitope, HA_91–108_. Its amino acid sequence was conserved at least in the nine H3 strains of IAV (16). However, when expressed alone on flagellin, the epitope only conferred partial protection to immunised mice challenged with IAV strain A/Texas/1/77 (H3N2) (17). Based on this finding, two conserved epitopes of IAV NP, NP_55–69_ (Th epitope) and NP_147–158_ (cytotoxic T lymphocyte [CTL] epitope), were then combined with the previously described B cell epitope to form a recombinant triepitope vaccine (18). The triepitope peptide vaccine provided greater protection than the single epitope. Moreover, the immune responses induced by the triepitope peptide vaccine lasted longer and were able to protect vaccinated mice challenged with influenza virus seven months since the last boost (18). Long-term protection was mostly provided by the Th epitope in the triepitope peptide vaccine.

Although the triepitope peptide vaccine induced long-lasting immunity, immune responses induced by NP_55–69_ and NP_147–158_ were major histocompatibility complex (MHC)-restricted (18). Immune responses to MHCrestricted epitopes depend greatly on the compatibility of the recipients’ MHC with the epitopes the higher the compatibility, the greater the responses; and vice versa (19). As a result, different individuals may respond differently to the triepitope peptide vaccine. To solve this problem, peptides representing four different conserved epitopes that matched better with Caucasian human leukocyte antigen (HLA) were synthesised: (i) B cell epitope, HA_91–108_; (ii) Th epitope, HA_307–319_ which is restricted to most HLA class II molecules (DR1, DR2, DR4, DR5, DR7, DR9 and DR52A); (iii) CTL epitope, NP_335–350_ which is restricted to HLA-A2, A3, Aw68.1 and B37 and (iv) CTL epitope, NP_380–393_ which is restricted to HLA-B8 and B27 (20). The newly selected epitopes can induce prominent protective and cross-strain (H1N1, H2N2 and H3N2) immunity when administered to Balb/C mice transplanted with human peripheral blood mononuclear cells (20). The anti-influenza peptides thus matched the criteria for universal influenza vaccines in terms of preventing infections by multiple influenza subtypes.

The other two influenza epitopes, namely, HA_354–372_, which is conserved in influenza B virus (14), and M1_2–12_, which is restricted to HLA-A2 and -A3 (15), were added to the four epitopes to form a polyepitope peptide vaccine composed of the six conserved epitopes of IAV. The vaccine induced cross-reactive antibodies and cellular antiviral responses that implicated T and NK cell activities and complement-mediated lysis. The protective effects were restricted not only to human influenza viruses but also to the avian influenza virus H5N1 (15).

**Figure 1:**
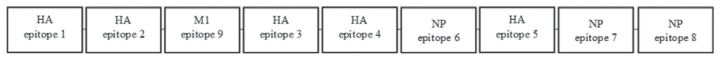
The arrangement of epitopes in M-001 vaccine. This formulation induced high levels of protection against various influenza strains in immunised animals

The final formulation of the polyepitope peptide vaccine consisted of nine epitopes (Figure 1, Table 1) and was termed as M-001 (11). The authors did not elaborate deliberately the rationale of incorporating additional three epitopes to the previous six conserved epitopes. The multi-epitope peptide vaccine was expressed in *Escherichia coli* and showed high level of protection against multiple influenza strains in animal studies (11). The vaccine has been subjected to human clinical trials as a result of its extraordinary immunogenicity.

In human trials, M-001 vaccine induced robust humoral and cell-mediated responses in young adults aged 18–49 years old (11). Humoral response was prominent when the immunisation of M-001 was paired with Montanide ISA 51 VG adjuvant. Humoral response was further enhanced by complement factors (11). The incubation of antiserum raised using M-001 vaccine with complement factors triggered the lysis of Madin-Darby Canine kidney (MDCK) cells infected with circulating H1N1, H3N2 and influenza B (Victoria lineages). In addition, the elevation of interleukin-2 (IL-2) and IFN-γ levels in the serum after M-001 immunisation confirmed cell-mediated immunity, especially in those subjects immunised with 500 μg of M-001 vaccine without adjuvant. Cytokines, such as IL-2 and IFN-γ, are key factors in immune cell differentiation and survival, regulation of immune functions and enhancement of antibody response (11). Besides young adults, the elderly (≥ 65 years old) showed promising immune responses three weeks after they were vaccinated with M-001 (21).

In a prime-boost immunisation, M-001 vaccine was used as a priming dose before seasonal trivalent influenza vaccine (TIV) immunisation. The prime-boost immunisation strategy triggered immunity against TIV strains and cross-immunity against heterologous influenza strains (21). Priming with M-001 prior to 2011/12 TIV resulted in a considerable increase in HA1-specific antibodies against influenza strains included in the 2011/12 TIV formulation and IAV strain A/Switzerland/9715293/2013, which caused an epidemic in the United States in 2014 and 2015 (21). Priming with M-001 expands helper T cell clones, which in turn promote the clonal expansion of B cells and increase HA1 antibody titre (22). The success of M-001 vaccine in the prime-boost approach has led to the proposition of large-scale human trials for the study of the immunogenicity of M-001 either as stand-alone vaccine or as priming dose for H5N1 influenza vaccine (23).

### FLU-v

FLU-v is a synthetic polyepitope peptide vaccine composed of conserved T cell epitopes that complement specifically with mouse MHC (H-2Kb) and human HLA (HLA-A*0201) (22). Initially, in an animal study, six peptides were included in the FLU-v formulation. Two of these peptides were derived from influenza M2 and PB1, whereas the other four peptides belonged to M1 and NP antigens (24). The peptides were selected based on the following criteria: (i) length must not be more than 40 amino acids; (ii) composed of at least five human T cell epitopes; and (iii) with a probability of 10^−10^ or less for any peptide not containing at least one of the identified T cell epitopes (24). One of the M1 and PB peptides were excluded in the clinical trials so that the high similarity of amino acid sequences (> 70%) amongst the peptides was ensured; hence, the selected peptides consistently contained > 5 human T cell epitopes (25). The final formulation of FLU-v vaccine is summarised in Table 2.

Unlike the currently licensed influenza vaccines that mainly target humoral immune response, FLU-v focuses on the induction of CD8+ T-lymphocytes. It was developed based on the fact that CD8+ CTL is responsible for virus clearance and recovery. Memory T cells protect individuals from influenza virus infections without relying on humoral immune response (26). Although current vaccines induce specific T cell responses, the magnitude is comparatively low, particularly amongst elderly population (27). Furthermore, current vaccines mainly induce immune responses against viral surface antigens, particularly HA and their effectiveness in stimulating the proliferation of IFN-γ-producing T cells in adult population is low. This observed phenomenon is attributed to the fact that most T cell epitopes are located in the internal proteins of influenza viruses, such as NP, M1 and polymerase subunits (PA, PB1 and PB2) (27).

An in vivo study (24) proved that FLU-v triggers murine and human T cell immunity. A significant increase in IFN-γ level was observed when splenocytes from FLU-v-immunised mice were co-cultured with HLA-A*0201-restricted human T1 cells and H-2Kb-restricted EL-4 cells (murine T lymphocytes) infected with H1N1 and H3N2 influenza viruses. Even at a low dose, the vaccine was demonstrated to induce CTL immune response which increased the survival of mice challenged with a lethal dose of influenza virus (24).

However, the findings in clinical trials were slightly different from that observed in the in vivo study as high-dose (500 μg) and low-dose (250 μg) vaccine preparations require adjuvants to induce cellular responses in the clinical trials (25). The responses of the subjects to the vaccine varied. The responses observed in most of the immunised individuals were not long lasting. In addition, a single dose of FLU-v at 500 μg with adjuvant was not sufficient to completely prevent viral shedding and reduce symptom score during the infection course (28). Increasing the vaccine dose or number of booster probably would have improved the anti-influenza immune responses. A phase IIb clinical trial evaluating immune response to different doses and formulations of FLU-v was then proposed (29). Despite the pending clinical findings of the phase IIb trial, FLU-v is believed to be a potential universal influenza vaccine because it induces a higher frequency of cellular responses than natural infections. This characteristic is mostly attributable to the presence of T cell epitopes derived from influenza internal antigens in FLU-v peptide vaccine formulation (27).

### Flunisyn^™^ (FP-01.1)

Peptide-based vaccines require immunostimulants or adjuvants to overcome their poor immunogenicity (13). Flunisyn^™^ or FP-01.1 is a chemically synthesised vaccine that consists of six different 35-mer conserved epitopes of influenza A NP, M, PB1 and PB2 proteins (30) (Table 3). These epitopes are HLA class-I and class-II restricted. In its formulation, each peptide is linked to a fluorocarbon chain [C_8_F_17_(CH_2_)_2_-COOH] for the enhancement of immunogenicity. Fluorocarbon chain helps prolong the exposure of peptides to the immune system by promoting the half-life of peptides, thereby enhancing immunogenicity (30). In addition, fluoropeptides are stable and resistant to proteolytic degradation. The binding of an eight-carbon chain to either the amino or carboxyl terminus induces greater CD4+ and CD8+ T cell responses in mice than native peptides (31).

FP-01.1 can provide cross-reactive immune responses against H1N1 and H3N2 influenza subtypes. This universal influenza vaccine candidate is especially effective in the elderly population; it significantly induces the production of IFN-γ, IL-2 and granzyme B in vaccinated persons (30). The elderly population is at high risk of having serious infection complications and have high mortality when infected by influenza viruses, but current influenza vaccines only confer limited protection (32). Granzyme B is an important effector in CTL-mediated immune responses, particularly in diminishing influenza-associated complications in the elderly (33). Hence, influenza vaccine, such as FP-01.1, which can stimulate granzyme B activity in influenza vaccination programme must be included. Such strategy is believed to promote virus eradication and recovery.

### Modified Vaccinia Ankara-based Influenza Vaccine

Modified vaccinia Ankara (MVA) is an attenuated vaccinia virus that has been shown to prime T cell responses towards the antigens they carry. Given its promising adjuvant effect, MVA has been used as a vaccine carrier for malaria, human immunodeficiency virus (HIV) and tuberculosis vaccines (34). MVA offers several advantages as vaccine carrier, such as: (i) excellent safety profile in children and HIV-positive individuals; (ii) great stability; (iii) rapid stimulation of humoral and cellular responses and (iv) various vaccine inoculation routes.

Several MVA-based influenza vaccines have been developed and are undergoing rigorous testing. Amongst them, MVA-NP+M1 vaccine has now reached the clinical trial phase (35). The complete NP and M1 antigens are expressed and displayed on MVA virus particles. Previously, in a preclinical study using a mouse model, MVA-NP+M1-immunised mice survived longer and better than the control group when challenged with heterologous PR8 (H1N1), X31 (H3N2) and H17 IAV (36). This vaccine was thus believed to be a potential universal vaccine candidate. It induces broad-spectrum and long-lasting immunity against a variety of influenza A sub-types via cross-reactive T cell immune responses. This characteristic is especially important to those formerly exposed to influenza infections because their memory T cell levels are generally insufficient to protect them from reinfections by influenza A viruses (34). As a result, MVA-NP+M1 can be an alternative to currently available trivalent inactivated and live attenuated influenza vaccines (37). In phase I clinical trial, the vaccine was shown to boost reactive T cell responses in adult volunteers. The levels of CD4+ and CD8+ T cell populations increased after MVA-NP+M1 vaccination, which increased the production of IFN-γ, IL-2, TNF-α and CD107a (34). These cytokines initiate and amplify cell-mediated immunity for influenza virus clearance and prevention of virus shedding.

Another MVA-based influenza vaccine that has reached the clinical trial phase is MVA-H5 clade 1 vaccine. Mice and macaques immunised with the vaccine were protected against homologous clade 1 avian influenza A virus (A/Vietnam/1194/04) and heterologous avian influenza A virus of clade 2.1 (A/Indonesia/5/05) (38, 39). The cross-clade protection was induced even at a low dose and after single vaccination. Two immunisations with low dose (10^4^ pfu) of MVA-H5 clade 1 vaccine were sufficient to provide protection against homologous and heterologous viruses. However, in any pandemic influenza event, two or more immunisation regimens might be difficult due to time constraint (40). As a result, single immunisation with high dose of vaccine (10^8^ pfu) was proposed. The single immunisation regime protected mice from severe disease symptoms caused by homologous and heterologous influenza strains (40). Interestingly, the antibodies raised by the vaccine showed promising neutralising activities against 13 H5 antigens from various clades. Additionally, antibody-dependent cellular cytotoxicity and T cell responses were also observed, but to a lesser extent (41).

## Immune Responses Induced by Conserved Influenza Antigens

One of the major challenges in developing universal influenza vaccines is choosing the most promising conserved epitopes that can induce the desired cross-protective immune responses (42). For example, the conserved stalk region of influenza hemagglutinin, HA2, induces humoral responses to neutralise influenza viruses (42), whereas some internal antigens carrying conserved epitopes, such as NP and M1, promote cellular immune responses that are responsible for viral clearance and speedy recovery (43). The latter was well demonstrated by the FLU-v vaccine.

The mechanisms of actions of anti-influenza antibodies have been documented. Neutralising antibodies normally target external epitopes, such as HA (44), whereas non-neutralising antibodies recognise and bind internal epitopes, such as those found on M2 and NP (45). By binding to HA epitopes, neutralising antibodies are capable of inhibiting virus attachment to host cell receptors or interfering with the conformational change of HA that is necessary to expose fusion peptides (44). However, non-neutralising antibodies mostly provide anti-influenza protection in association with cytotoxic mechanisms, such as antibody-dependent cellular phagocytosis (ADCP) and complement-mediated antibody-dependent cellular cytotoxicity (ADCC) (45). M2 protein is expressed abundantly on host cell membranes, which in turn allow the recognition and binding of M2-specific antibodies. The Fc portions of the antibodies then bind to the Fc receptors of natural killer cells to initiate the ADCP and ADCC of infected cells (46). Anti-NP IgG also plays a pivotal role in heterosubtypic immunity. The non-neutralising anti-NP antibodies inhibit viral RNA synthesis and the translocation of ribonucleoprotein complex through the actions of ADCC and antibody-mediated cytokine induction (45, 47).

Besides humoral responses, many viral vaccines target virus-specific cell-mediated immunity (CMI), especially CD4+ T helper and CD8+ T cells (48). In influenza vaccination, CMI removes virally infected cells and overcomes the limitations of humoral responses caused by gene mutations in the surface antigens of circulating influenza strains (49). Influenza-associated CMI typically targets the internal antigens of influenza viruses, such as NP, PB1 and M1, which are far less susceptible to antigenic changes (50, 51). Upon influenza virus infections, CD8+ T cells recognise the influenza peptides associated with MHC class I molecules and subsequently differentiate into effector CTLs under the influence of pro-inflammatory cytokines, such as IFN-γ, interleukin (IL)-2 and IL-12 (52). CTLs then mediate the lysis of virus-infected cells and produce inflammatory cytokines to restrict viral replication; altogether, this process reduces the viral load and the length of infection (49). CTL-induced cell lysis is mediated by cytotoxic granules known as perforin and granzymes. Perforin binds to target cell membranes to form pores which then allow entry of granzymes to induce and initiate apoptosis of infected cells (52). Meanwhile, CD4+ helper T cells recognise the influenza peptides presented on MHC class II molecules on antigen presenting cells, such as B cells, macrophages and dendritic cells. Activated CD4+ T cells subsequently differentiate into: (i) Th1 cells that produce proinflammatory cytokines, such as IFN-γ and IL-2, which participate effectively in CMI and (ii) Th2 cells that produce IL-4 and IL-13 to mediate activation and proliferation of B cells into antibody-producing plasma cells (52).

Previous studies underscored the importance of both humoral immunity and CMI in conferring effective immune protection against heterologous influenza viruses (49, 53). Hence, the idea of incorporating a number of conserved influenza peptides, especially those derived from internal viral antigens in a single universal influenza vaccine formulation, has been endeavoured in the studies described in this review. These vaccine candidates are expected to confer long-term memory immunity against subsequent influenza virus infections. This occurrence is particularly important to overcome the issue of short-lived influenza-specific antibody titres induced by presently available influenza vaccines. Influenza-specific IgM and IgG induced through either vaccination or natural influenza can only be sustained up to 18 months. Meanwhile, the memory CD4+ and CD8+ T cell-mediated immunity initiated by influenza vaccination can only be sustained at a relatively high level of up to 6 months and followed by a sharp decline within 12 months (54).

## Multi-Epitope-Based Vaccines versus MVA-Vectored Vaccine

The discovery of immunogenic epitopes in influenza antigens has led to the evolution of influenza vaccine formulation to epitope-based vaccines. Multi-epitope-based vaccines are developed to imitate natural influenza infections in which influenza viruses carry multiple antigens on virus particles. Multi-epitope-based vaccines can therefore induce high-level immunity that can safeguard recipients from subsequent influenza virus invasion. Immune responses induced by multi-epitope-based vaccines can promote premature virus degradation intracellularly and enhance virus recognition by immune cells (55).

MVA is a replication-deficient viral vector that is usually harnessed to display foreign antigens. Its non-replicating characteristic makes it well tolerated and safe to be administered; its safety feature has been demonstrated in immunodeficient animal models (46). Moreover, its intrinsic adjuvanticity mediated by Toll-like receptors and inflammasome adds to its benefits as a prominent vaccine carrier cargo. As a result, the immunogenicity of foreign antigen presented on MVA vector is greatly enhanced (46) which is similar to that observed in MVA smallpox vaccine produced for emergency use in the United States (56). The large-scale manufacturing process of vaccinia-based vaccines has been established using chick embryo fibroblasts. Previously, in the preparation of MVA smallpox vaccine in the United States, approximately 20 million doses of MVA smallpox vaccine were produced (56), which is beneficial for the mass production of influenza vaccines to counter the upcoming influenza outbreaks.

## Conclusion

Immune responses raised against homologous and heterologous influenza viruses using universal influenza vaccines largely depend on the immunogenicity of conserved epitopes included in vaccine formulations. Despite the limitations, such as low immunogenicity associated with peptide vaccines, the combination of several promising conserved epitopes in a single peptide helps address the immunogenicity issue. An effective vaccine is expected to stimulate both arms of the immune system. A few polyepitope peptide vaccines were proven to prime humoral and cellular immunities against homologous and heterologous influenza subtypes. The cross-reactive antibodies prevent the entry of influenza viruses, help slow down viral infection processes and accelerate virus clearance by inducing complement-dependent or antibody-dependent cell cytotoxicity. Cell-mediated immunity helps reduce the severity of illnesses by inhibiting virus reproduction and spread.

Although universal influenza vaccines are expected to protect immunised individuals against all or most influenza types, emphasis is increasingly given to the prevention of influenza A infections. Such infections are crucial public health issues worldwide and cause significant morbidity and mortality. An ideal universal influenza vaccine should also provide durable protection in all age groups, including high-risk and immunocompromised individuals. A protection duration of 5–10 years is preferable but a 12-month protection period is mandatory to cover a full season of influenza. Conclusively, the use of universal influenza vaccines overcomes the limitations associated with the current influenza vaccines and promises an effective solution for future influenza pandemic caused by unexpected influenza virus strains.

## Figures and Tables

**Table 1:** Formulation of M-001 peptide vaccine

Peptides	Amino acid sequences	T or B cell epitope
HA epitope 1	PKYVKQNTLKLAT	Th
HA epitope 2	SKAYSNCYPYDVPDYASL	B
HA epitope 3	WLTGKNGLYP	B
HA epitope 4	WTGVTQN	B
HA epitope 5	PAKLLKERGFFGAIAGFLE	B
NP epitope 6	FWRGENGRKTRSAYERMCNILKGK	Th
NP epitope 7	SAAFEDLRVLSFIRGY	CTL
NP epitope 8	ELRSRYWAIRTRSG	CTL
M epitope 9	SLLTEVETYVP	B and CTL

**Table 2:** Formulation of FLU-v vaccine

Antigens	Amino acid sequences
M1A^a^	DLEALMEWLKTRPILSPLTKGILGFVFTLTVP (32 aa)
NPA^a^	DLIFLARSALILRGSVAHKSC (21 aa)
NPB^b^	PGIADIEDLTLLARSMVVVR (20 aa)
M2^c^	IIGILHLILWILDRLFFKCIYRLF (24 aa)

Notes:

a Amino acid sequence corresponds to the consensus sequence of M1 and NP of influenza A;

b amino acid sequence corresponds to the consensus sequence of NP of influenza B;

c amino acid sequence corresponds to the consensus sequences of M2 of influenza A and B

**Table 3:** Peptide sequences of Flunisyn^™^ (FP-01.1)

Peptides	Amino acid sequences	Internal proteins
P44	HMAIIKKYTSGRQEKNPSLRMKWMMAMKYPITADK	PB2
P220	VAYMLERELVRKTRFLPVAGGTSSVYIEVLHLTQG	PB2
P1100a	YITRNQPEWFRNVLSIAPIMFSNKMARLGKGYMFE	PB1
P1116b	APIMFSNKMARLGKGYMFESKRMKLRTQIPAEMLA	PB1
P3071	DQVRESRNPGNAEIEDLIFLARSALILRGSVAHKS	NP
P3845	DLEALMEWLKTRPILSPLTKGILGFVFTLTVPSER	M1
